# Supplementary material for: An updated analysis of opioids increasing the risk of fractures
Source: PLoS One. 2020 Apr 9;15(4):e0220216. doi: 10.1371/journal.pone.0220216 (PMC7145014; doi:10.1371/journal.pone.0220216)
Supplement: S1 File — (PDF) [file pone.0220216.s002.pdf]

AJE®

Editing Certificate

This document certifies that the manuscript

An update analysis of Opioids increase the risk of fractures

prepared by the authors

Qiaoning Yue, Zhaowei Teng

was edited for proper English language, grammar, punctuation, spelling, and overall style by one or more of the highly qualified native English speaking editors at AJE.

This certificate was issued on **October 22, 2019** and may be verifed on the AJE website using the verification code **62A0-65F4-F055-6FAF-254P**.

Neither the research content nor the authors' intentions were altered in any way during the editing process. Documents receiving this certification should be English-ready for publication; however, the author has the ability to accept or reject our suggestions and changes. To verify the final AJE edited version, please visit our verification page at aje.com/certificate. If you have any questions or concerns about this edited document, please contact AJE at support@aje.com.

AJE provides a range of editing, translation, and manuscript services for researchers and publishers around the world. For more information about our company, services, and partner discounts, please visit aje.com.
