# Supplementary material for: An updated analysis of opioids increasing the risk of fractures
Source: PLoS One. 2020 Apr 9;15(4):e0220216. doi: 10.1371/journal.pone.0220216 (PMC7145014; doi:10.1371/journal.pone.0220216)
Supplement: S1 Data — (DOC) [file pone.0220216.s003.doc]

| Author,  year,  location | Age,  years | Fracture type/assessment | Study design | Sample size | Follow-up time | Models | Adjustment for covariates | NOS | RR | 95%CI |
| --- | --- | --- | --- | --- | --- | --- | --- | --- | --- | --- |
| Jensen, 1991, Denmark[34] | >59 | Hip fracture/WHO code 820 | Case-control | 400 | From April to December 1988 | Cornfield’s iterative method | Age, sex, nursing home residency and number of hospital admissions | 6 | 1 | （0.5-1.98） |
| Shorr, 1992, Canada[22] | ≥65 | Hip fracture/ ICD-8 and ICD-9 | Case-control | 28541 | From 1997 to 1985 | Unconditional logistic regression | Age, sex, home, hospital discharge in the preceding year, and index year | 7 | 1.6 | （1.4-1.9） |
| Guo, 1998, Sweden[23] | ≥75 | Hip fracture/ICD-9 | Prospective cohort | 1608 | 4.4 years | Cox proportional hazards | Age, sex, education, residence, ADL limitations,cognitive impairment, and history of stroke and tumors | 8 | 2.01 | （1.19-3.4） |
| Ensrud, 2003, USA[24] | ≥65 | Fractures/ radiology reports | Prospective cohort | 8127 | 4.8 years | Cox proportional hazards | Age, sex, race, health status, smoking, walking exercise,functional impairment, cognitive function,depression, and weight change | 9 | 1.4 | （1.06-1.83） |
| Card, 2004, UK[25] | NA | Hip fracture/NA | Prospective  cohort | 99467 | 7.3 per 10000 person-years | Cox regression | Age, sex, practice, and corticosteroid use | 6 | 1.67 | （1.12-2.48） |
| Sachin, 2006, USA[26] | ≥65 | Hip fracture/ICD-9 | Prospective cohort | 362503 | 464 days | Cox regression | Age, sex, and use of antidepressants, antipsychotics, and anxiolytics/hypnotics | 7 | 2.05 | （1.87-2.25） |
| Vestergaard, 2006, Denmark[6] | 43.44 ± 27.39 | Hip fracture/NA | Case-control | 42065 | During 2006 | Conditional logistic regression | Use of other drugs | 6 | 1.26 | （1.11-1.43） |
| Kathleen, 2010, USA[15] | ≥60 | Fractures/ ICD-9 | Prospective  cohort | 2341 | 32.7 months | Cox proportional  hazards | Age, gender, smoking, depression, substance abuse, dementia, comorbidity,prior fracture, pain site, antidepressant use, sedative use, and HRT/bisphosphonate use | 9 | 1.28 | （0.99-1.64） |
| Miller, 2011, USA[27] | ≥65 | Fractures/ ICD-9 | Retrospective cohort | 17310 | 451 per 1000 person-years | Cox proportional  hazards | Age,sex,diabetes,stroke,osteoarthritis,comorbidity index, stroke, diabetes | 6 | 4.9 | （3.5-6.9） |
| Vestergaard, 2012, Denmark[28] | 45 to 58 | Fractures /X-ray | Prospective  cohort | 2016 | 10 years | Cox proportional  hazards | Age, HT, BMI, baseline spine bone mineral density (BMD), family or prior fracture, history serum 25-hydroxy-vitamin levels and smoking | 9 | 1.49 | （0.97-2.31） |
| Laura, 2013, USA[29] | ≥58.73 ±13.43 | Lower extremity /ICD-9 | Retrospective  cohort | 7447 | 3-8 years | Cox proportional  hazards | Age, race, completeness of spinal cord injury (SCI) level and duration of SCI | 7 | 1.82 | （1.59-2.09） |
| Lin Li, 2013, UK[30] | 18 to 80 | Fracture/NA | Nested case-control | 71538 | From 1990 to 2008 | Conditional  logistic regression | Smoking, BMI, comorbidities, and Number of general practice visits recorded during the years before index date | 7 | 1.27 | （1.21-1.33） |
| Kristine, 2014, Sweden[32] | ≥75 | Hip fracture/codes S72.0,  S72.1, and  S72.2 | Retrospective cohort | 38407 | During 2006 | Multivariate logistic regression | Age, gender and morbidity level | 8 | 1.56 | （1.43-1.82） |
| Leach, 2015, Australia[33] | >65 | Hip fracture/ICD codes S72.0 or S72.1 | Case-crossover | 8828 | From 2009 to 2012 | Conditional logistic regression | NA | 8 | 1.62 | （1.42-1.84） |
| Acurcio, 2016, Canada[31] | 76.33 ±10.04 | Fracture/ICD-9,  ICD-10 | Retrospective nested case-control | 9769 | From 2007 to 2012 | Conditional  logistic  regression | Age, sex, measures of comorbidities, history of arthroplasty, use of corticosteroids use, biologic agents or traditional disease-modifying antirheumatic drugs (DMARDs), use of other drugs potentially influencing the risk of fractures or falls, and measures of health care resource use | 7 | 2.89 | （2.49-3.38） |
| Grewal, 2018, Canada[12] | ≥65 | Fracture/ICD-10 | Retrospective  cohort | 89897 | 3 months | Cox regression | Age, sex, past medical history, health care use, etc. | 7 | 3.59 | （1.97-6.13） |
| Taipale, 2018, Finland[10] | NA | Hip fracture/ ICD-10 | Retrospective  matched cohort | 70718 | 5 years | Cox proportional hazard | Age, sex, time since Alzheimer's disease(AD) diagnosis, socioeconomic status, university hospital department area, use of drugs, and comorbidities | 9 | 1.96 | （1.27-3.02） |
| Vakharia, 2019, USA[35] | ≥64 | Fracture/ICD-9 (81.54) codes 304.00-304.02 and 305.50-305.52 | Retrospective matched cohort | 23072 | From 2005 to 2014 | R Statistical analysis | Age, sex, and use of drugs | 7 | 1.83 | （1.16-4.79） |
